# Supplementary material for: The X-linked splicing regulator MBNL3 has been co-opted to restrict placental growth in eutherians
Source: PLoS Biol. 2022 Apr 27;20(4):e3001615. doi: 10.1371/journal.pbio.3001615 (PMC9084524; doi:10.1371/journal.pbio.3001615)
Supplement: S1 Text — (PDF) [file pbio.3001615.s001.pdf]

Supplementary Discussion

Emergence of the short MBNL3 isoform

Intricately linked to placental recruitment of *Mbnl3* has been the emergence of a short isoform lacking the first zinc finger pair. Whilst alternatively spliced transcripts with the potential to code for a short isoform have been described for *Mbnl1* and *Mbnl2*, these appear to be poorly translated and to produce unstable proteins [1-3]. Indeed, production of these alternative transcripts has been proposed to act as a mechanism by which *Mbnl* genes can limit their own production, as the AS event generating them is auto-regulated by MBNL proteins [1, 4]. Therefore, the production of a stable and highly expressed short protein isoform lacking the first zinc finger pair is unique to eutherian *Mbnl3*. This further suggests that some of the amino acid changes seen in eutherian *Mbnl3* may be linked to stabilization of the truncated protein and/or enhanced short isoform translation.

The short isoform in placenta is mainly generated through the use of a novel eutherian-specific transcription start site. Interestingly, this transcription start site is closely associated with two eutherian-specific transposable elements of the MER53 and MER103c families, which are ~200 and ~850 bps away from the eutherian-specific TSS, respectively, and whose sequence appear largely conserved across this lineage. Given that transposable elements have been repeatedly shown to provide the raw evolutionary material for the evolution of new enhancers and transcription start sites [5, 6], it is tempting to speculate their insertion in eutherian ancestors might be associated to the origin of the short MBNL3 isoform in placenta.

Evolution of the binding preferences of MBNL3 isoforms

The majority of full-length MBNL proteins, including MBNL1 and MBNL2 from all tested species and MBNL3 from non-eutherian species, have a strong GCUU binding preference (this paper and [7-10]). In contrast, we found the long isoform of mouse Mbnl3 alongside the majority of short MBNL isoforms tested, including mouse MBNL3, to preferentially bind a GCA/U motif and show weaker binding preferences. Previous studies have suggested the GCUU binding preference of canonical MBNL proteins is largely driven by the first zinc finger pair and in particular ZF2 [11-13]. This is in keeping with our results for the short MBNL isoforms, which lack this zinc finger pair, and suggests the binding preference changes seen for full-length eutherian MBNL3 vs. other MBNL proteins could be due to either a switch in dominance between the zinc fingers and/or changes in the binding preferences of ZF2.

Furthermore, it raises the possibility that the that initial RNA interaction event(s) that mediated reduced placental growth were mediated by the second zinc finger pair and that there was then subsequent selection for mutations that favored these interactions. Our chimeric experiments revealed the binding preference of mouse MBNL3 are determined by both the zinc finger domains, which are known to mediate binding, and the disordered linker region between the zinc finger domains. The precise function of the linker in altering the binding preference of MBNL proteins is unclear; however, there is a growing pool of evidence that disordered regions can directly bind RNA and may cooperate with globular RNA motifs in this process [14]. Indeed, previous studies have found the linker to play an important role in RNA binding [15-17]. Alternatively, the linker could directly or indirectly regulate the accessibility/availability of individual zinc-fingers for binding.

The role of Mbnl2 *in placental growth regulation*

The effect of *Mbnl2* KO on placental growth appears to be at least in part indirect, as a reduction in placenta size is still seen when *Mbnl2* is specifically knocked out from epiblast but not trophoblast derived tissues (Fig. S5J). Whilst this may seem initially surprising, signalling from the epiblast and its derivatives is known to play important roles in regulating trophoblast growth. Specifically, epiblast derived Fgf4 and Nodal are required for Trophoblast stem cell maintenance at early post implantation stages [18] and, later in gestation, Igf2 from epiblast-derived tissues appears to be required for proper placental growth and development [19].

The link between the reduced placental size seen in both full and epiblast specific *Mbnl2* KOs and the reduced embryo size is unclear. The reduced placenta size appeared to precede the reduced embryo size, at least in the full KOs, suggesting it may be causative, as it has been shown that a reduction in placenta size can be compensated for by increased placental efficiency during early but not late gestation [20]. However, given that the embryo-derived factor causing the reduced placental growth in *Mbnl2* KOs must be almost certainly secreted and diffusible, it could also be expected to act on the tissues of the embryo proper, perhaps helping to match placental and foetal growth.

References

1. Konieczny P, Stepniak-Konieczna E, Taylor K, Sznajder LJ, Sobczak K. Autoregulation of MBNL1 function by exon 1 exclusion from MBNL1 transcript. Nucleic Acids Res. 2017;45:1760-75.

2. Kanadia RN, Johnstone KA, Mankodi A, Lungu C, Thornton CA, Esson D, et al. A muscleblind knockout model for myotonic dystrophy. Science. 2003;302(5652):1978-80.
3. Charizanis K, Lee K, Batra R, Goodwin M, Zhang C, Yuan Y, et al. Muscleblind-like 2-mediated alternative splicing in the developing brain and dysregulation in myotonic dystrophy. Neuron. 2012;75:437-50.
4. Konieczny P, Stepniak-Konieczna E, Sobczak K. MBNL expression in autoregulatory feedback loops. RNA Biol. 2018;15(1):1-8. Epub 2017/09/28. doi: 10.1080/15476286.2017.1384119. PubMed PMID: 28949831; PubMed Central PMCID: PMCPMC5786016.
5. Chuong EB, Elde NC, Feschotte C. Regulatory activities of transposable elements: from conflicts to benefits. Nat Rev Genet. 2017;18(2):71-86. Epub 2016/11/22. doi: 10.1038/nrg.2016.139. PubMed PMID: 27867194; PubMed Central PMCID: PMCPMC5498291.
6. Chuong EB, Rumi MA, Soares MJ, Baker JC. Endogenous retroviruses function as species-specific enhancer elements in the placenta. Nat Genet. 2013;45(3):325-9. Epub 2013/02/12. doi: 10.1038/ng.2553. PubMed PMID: 23396136; PubMed Central PMCID: PMCPMC3789077.
7. Goers ES, Purcell J, Voelker RB, Gates DP, Berglund JA. MBNL1 binds GC motifs embedded in pyrimidines to regulate alternative splicing. Nucleic Acids Res. 2010;38:2467-84.
8. Lambert N, Robertson A, Jangi M, McGeary S, Sharp PA, Burge CB. RNA Bind-n-Seq: quantitative assessment of the sequence and structural binding specificity of RNA binding proteins. Mol Cell. 2014;54:887-900.
9. Sznajder LJ, Michalak M, Taylor K, Cywoniuk P, Kabza M, Wojtkowiak-Szlachcic A, et al. Mechanistic determinants of MBNL activity. Nucleic Acids Res. 2016;44:10326-42.
10. Ray D, Kazan H, Cook KB, Weirauch MT, Najafabadi HS, Li X, et al. A compendium of RNA-binding motifs for decoding gene regulation. Nature. 2013;499:172-7.
11. Hale MA, Richardson JI, Day RC, McConnell OL, Arboleda J, Wang ET, et al. An engineered RNA binding protein with improved splicing regulation. Nucleic Acids Res. 2018;46:3152-68.
12. Purcell J, Oddo JC, Wang ET, Berglund JA. Combinatorial mutagenesis of MBNL1 zinc fingers elucidates distinct classes of regulatory events. Mol Cell Biol. 2012;32(20):4155-67. Epub 2012/08/15. doi: 10.1128/MCB.00274-12. PubMed PMID: 22890842; PubMed Central PMCID: PMCPMC3457334.
13. Park S, Phukan PD, Zeeb M, Martinez-Yamout MA, Dyson HJ, Wright PE. Structural Basis for Interaction of the Tandem Zinc Finger Domains of Human Muscleblind with Cognate RNA from Human Cardiac Troponin T. Biochemistry. 2017;56:4154-68.
14. Jarvelin AI, Noerenberg M, Davis I, Castello A. The new (dis)order in RNA regulation. Cell Commun Signal. 2016;14:9.
15. Tran H, Gourrier N, Lemercier-Neuillet C, Dhaenens C, Vautrin A, Fernandez-Gomez FJ, et al. Analysis of exonic regions involved in nuclear localization, splicing activity, and dimerization of Muscleblind-like-1 isoforms. J Biol Chem. 2011;286:16435-46.
16. Grammatikakis I, Goo Y, Echeverria GV, Cooper TA. Identification of MBNL1 and MBNL3 domains required for splicing activation and repression. Nucleic Acids Res. 2011;39:2769-80.
17. Kino Y, Mori D, Oma Y, Takeshita Y, Sasagawa N, Ishiura S. Muscleblind protein, MBNL1/EXP, binds specifically to CHHG repeats. Hum Mol Genet. 2004;13:495-507.
18. Guzman-Ayala M, Ben-Haim N, Beck S, Constam DB. Nodal protein processing and fibroblast growth factor 4 synergize to maintain a trophoblast stem cell microenvironment. Proc Natl Acad Sci U S A. 2004;101(44):15656-60. Epub 2004/10/27. doi:

10.1073/pnas.0405429101. PubMed PMID: 15505202; PubMed Central PMCID: PMCPMC524845.
19. Sandovici I, Georgopoulou A, Perez-Garcia V, Hufnagel A, Lopez-Tello J, Lam BYH, et al. The imprinted Igf2-Igf2r axis is critical for matching placental microvasculature expansion to fetal growth. Dev Cell. 2022;57(1):63-79 e8. Epub 2021/12/29. doi: 10.1016/j.devcel.2021.12.005. PubMed PMID: 34963058; PubMed Central PMCID: PMCPMC8751640.
20. Constancia M, Hemberger M, Hughes J, Dean W, Ferguson-Smith A, Fundele R, et al. Placental-specific IGF-II is a major modulator of placental and fetal growth. Nature. 2002;417(6892):945-8. Epub 2002/06/28. doi: 10.1038/nature00819. PubMed PMID: 12087403.
