# Supplementary figures and images for: The X-linked splicing regulator MBNL3 has been co-opted to restrict placental growth in eutherians
Source: PLoS Biol. 2022 Apr 27;20(4):e3001615. doi: 10.1371/journal.pbio.3001615 (PMC9084524; doi:10.1371/journal.pbio.3001615)

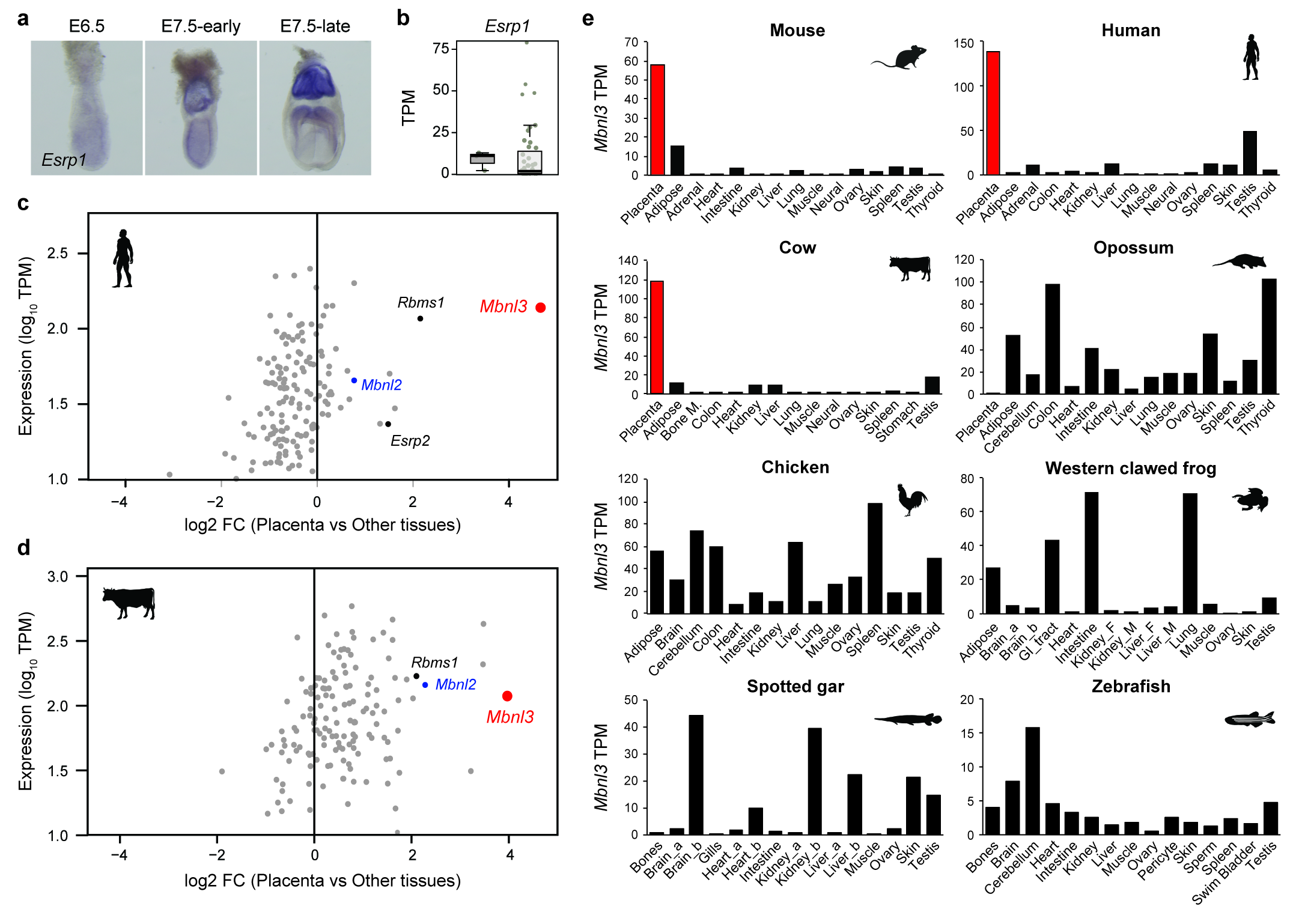

Supplement: S1 Fig — (A) Whole-mount in situ hybridisation analysis of Esrp1 in mouse embryos at the indicated stages. (B) Box plot showing expression levels of Esrp1 in placenta and nonplacental tissues in mouse. (C, D) Scatter plots showing placental expression level and placenta versus other tissues enrichment for 197 splicing regulators in human (C) and in cow (D). (E) Expression of Mbnl3 orthologs from different vertebrate species across differentiated adult tissues. Placenta samples are highlighted in red. RNA-seq samples are listed in S1 Table. The numerical data underlying this figure can be found in S1 Data. RNA-seq, RNA sequencing. (TIF) [file pbio.3001615.s002.tif]

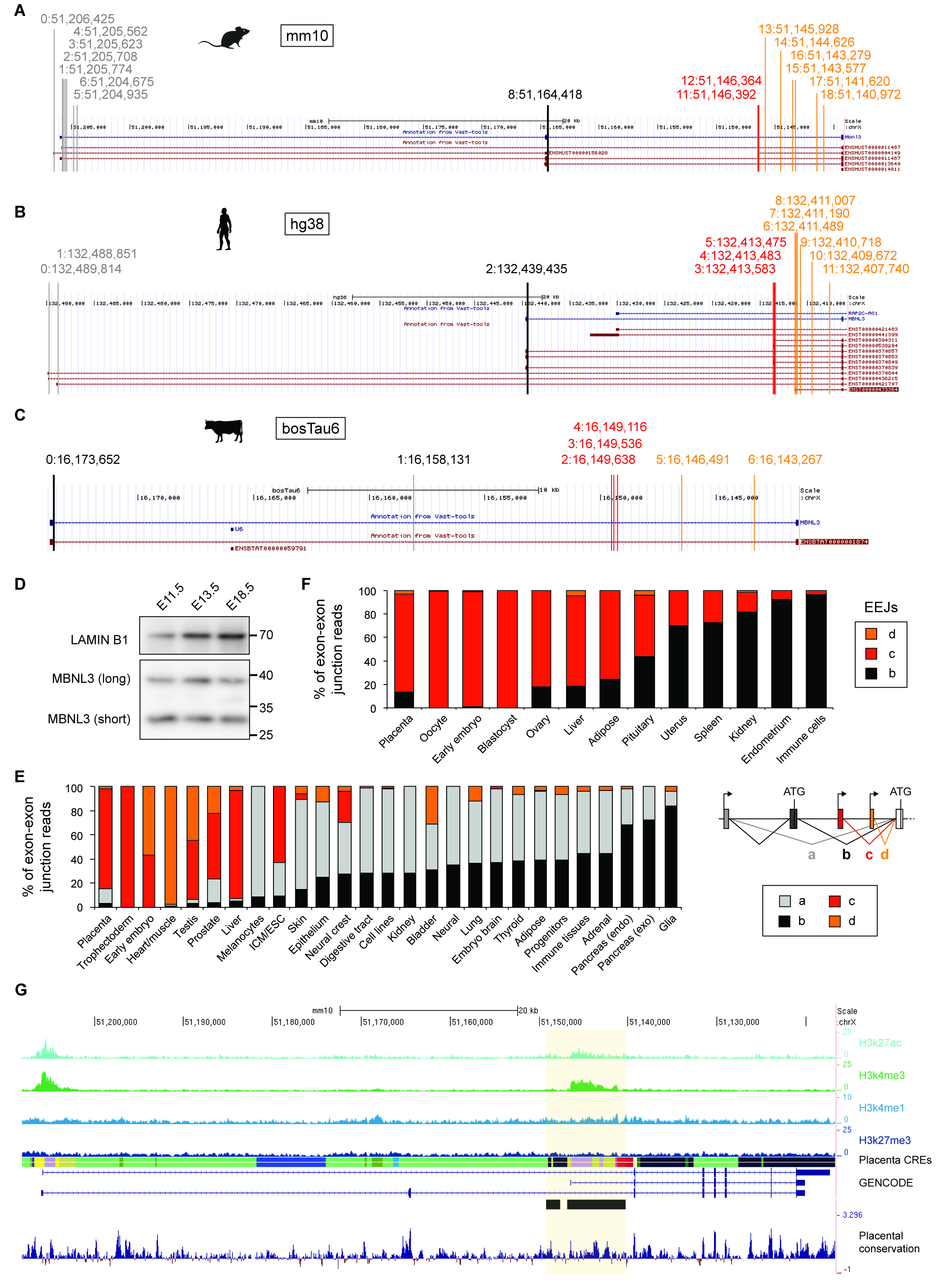

Supplement: S2 Fig — (A–C) Genome organization for mouse (A), human (B), and cow (C) of the annotated donor splice sites upstream of the second coding exon of Mbnl3, which encodes the internal ATG used to translate the short isoform. The coordinate on the X chromosome is indicated for each competing donor. The color code corresponds to that of Fig 2A, and the donor IDs correspond to that of S6 Table. (D) Western blot showing short (approximately 27 kDa) and long (approximately 38 kDa) MBNL3 isoforms in placenta at different developmental time points. The blot was conducted using an antisera raised against a 15-amino acid carboxyl-terminal MBNL3 peptide [21]. (E, F) Percent of exon–exon junction reads from each group of competing donors (as shown in A–C and schematized in the legend) across different samples from human (E) and cow (F). Individual donor counts for each sample are listed in S6 Table. (G) UCSC Genome Browser view of the mouse Mbnl3 genomic locus showing the injected promoter region (black box—yellow highlight). Top 5 tracts generated by Starks and colleagues [22] using E9.5 placenta ChIP-seq data. Track 5 (“Placenta CREs”) shows ChromHMM-defined chromatin states, including repressed regions (light blue), poised enhancers (dark green), active enhancers (red), and active promoters (light purple). Note, the injected promoter region was cloned as 3 fragments (see Methods) that were then ligated together to make the final tested construct; a portion of the locus was not included in this construct due to low levels of conservation with other placental mammals as indicated by the gap in the black box. The numerical data underlying this figure can be found in S1 Data. MBNL, Muscleblind-like; TSS, transcription start site. (TIF) [file pbio.3001615.s003.tif]

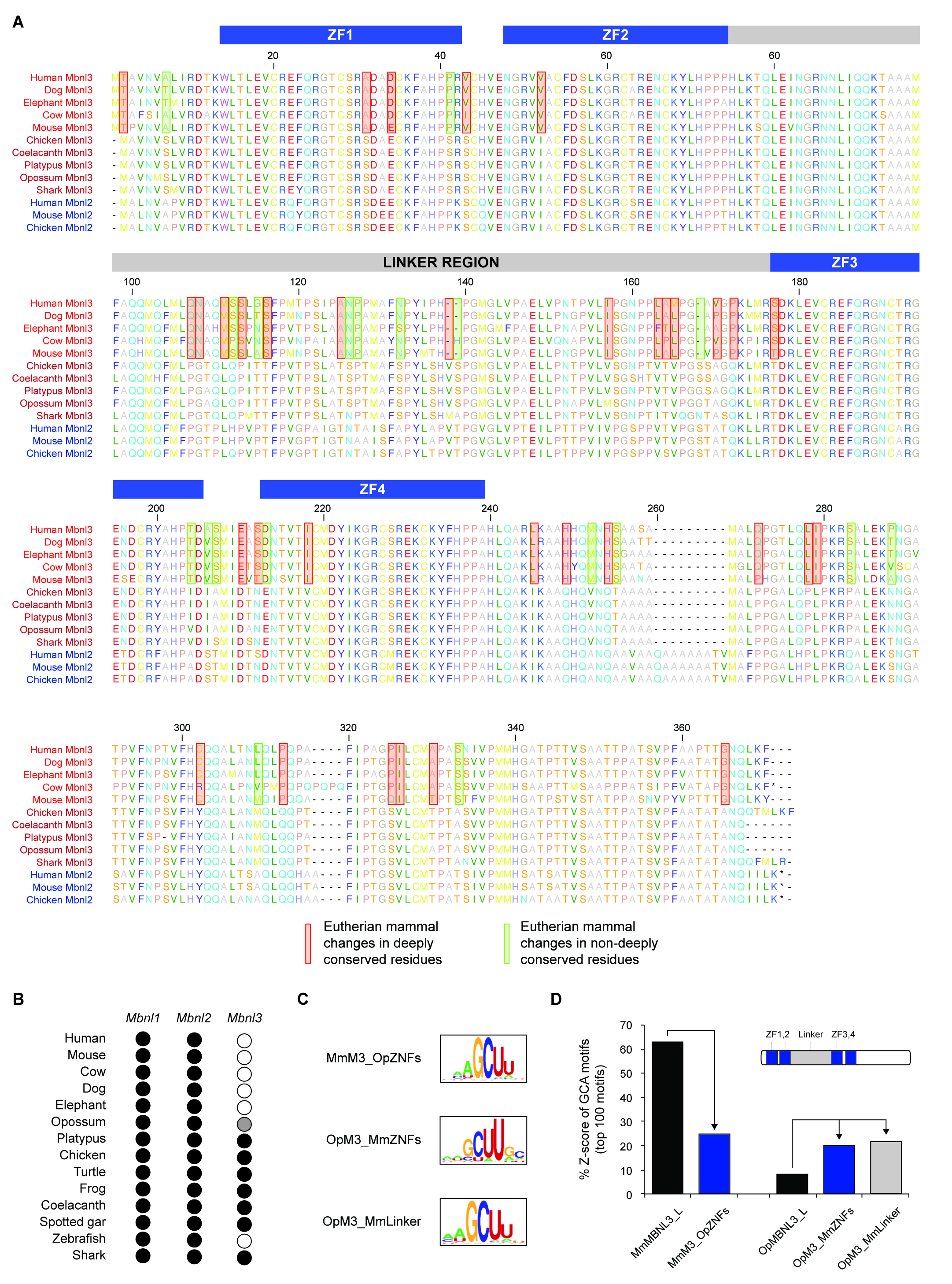

Supplement: S3 Fig — (A) Sequence alignment for different MBNL3 (red) and MBNL2 (blue) proteins from various vertebrate species. Eutherian changes in deeply conserved (conserved between MBNL2 and noneutherian MBNL3 proteins) and nondeeply conserved residues are highlighted using red and green boxes, respectively. ZF and the liker region are indicated. (B) Presence (black) or absence (white) of exon 5 in different Mbnl genes across vertebrates. In opossum, exon 5 can be identified, but it contains an in-frame stop codon (gray). (C) RNAcompete-derived sequence logos for the indicated chimeric proteins, with either mouse (Mm) or opossum (Op) ZF or linker regions in the other species’ backbone. (D) Analysis of the Z-score contribution of sequences containing GCA motifs to the total cumulative Z-score of the top 100 RNAcompete-derived 7-mers for the indicated WT and chimeric MBNL proteins. The numerical data underlying this figure can be found in S1 Data. MBNL, Muscleblind-like; WT, wild-type; ZF, zinc finger. (TIF) [file pbio.3001615.s004.tif]

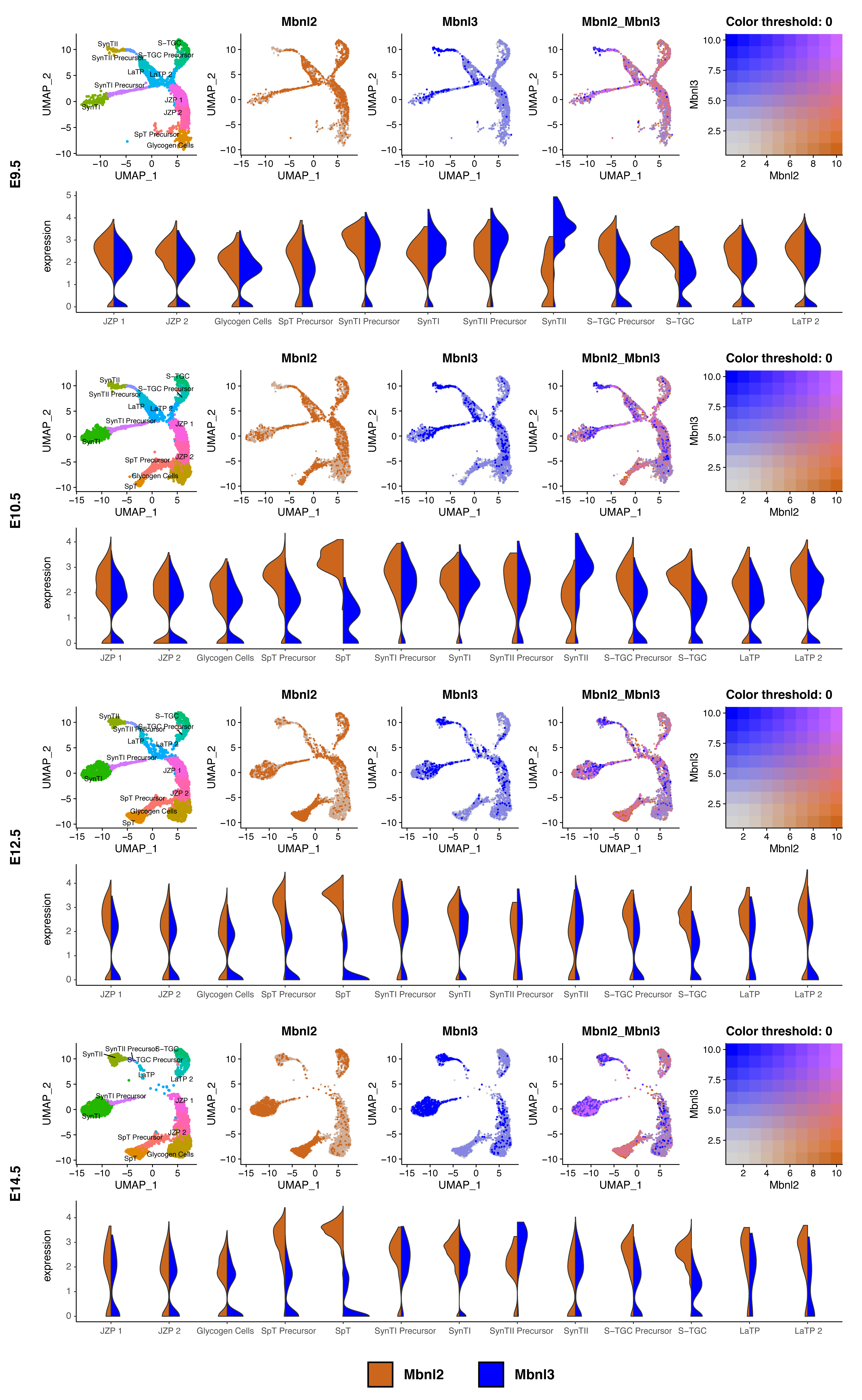

Supplement: S4 Fig — For each developmental stage, top, UMAP plots showing the distribution of cells across clusters (left) and the expression of Mbnl2, Mbnl3, and their co-expression in individual cells (right) obtained from [23]. The color code of the cells in the co-expression plot is defined according to the expression level of Mbnl2 and Mbnl3 as displayed in the color scale. Bottom, split violin plots showing the expression distribution of Mbnl2 and Mbnl3 for each cell cluster. The numerical data underlying this figure can be found in S1 Data. JZP 1/2, Junctional zone precursors subtype 1 and 2; LaTP, labyrinth trophoblast progenitor; LaTP 2, labyrinth trophoblast progenitor subtype 2; SpT, spongiotrophoblast; SynTI, syncytiotrophoblast layer 1; SynTII, syncytiotrophoblast layer 2; S−TGC, sinusoidal trophoblast giant cells. (TIF) [file pbio.3001615.s005.tif]

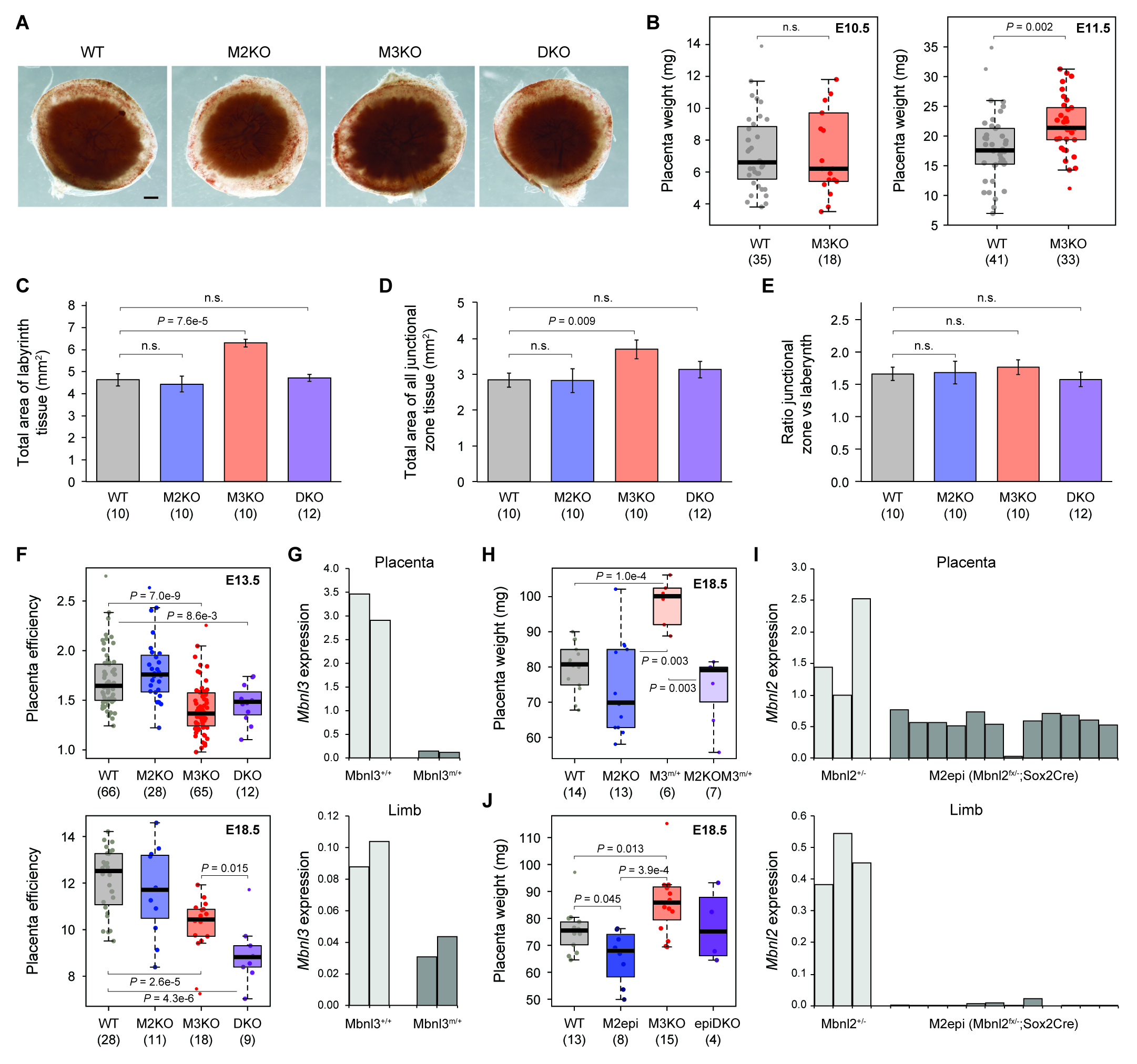

Supplement: S5 Fig — (A) Representative images of whole placentas at E18.5 for the indicated genotypes. (B) Effect of M3KO on placental weight at E10.5 and E11.5. (C–E) Quantification of the total area of labyrinth tissue (C), total area of junctional zone tissue (D) and ratio between labyrinth and junctional zone tissues for the indicated genotypes. (F) Effect of M2KO, M3KO or Mbnl2:Mbnl3 DKO on placenta efficiency (embryo weight/placenta weight) at E13.5 (top) and E18.5 (bottom). (G, I) Mbnl3 (F) and Mbnl2 (G) expression quantified by qPCR in the indicated genotypes. Each bar corresponds to a single placenta/limb sample. (H) Effects on placenta weight of placenta specific Mbnl3 knockout with (M2KOM3m/+) or without (M3m/+) universal M2KO. Note, some of these data points are also plotted in Fig 3A. (J) Effects on placenta weight of epiblast/fetus specific M2KO with (epiDKO) or without (M2epi) universal Mbnl3 knockout. For (B–F, H, and J), the number of placentas/embryos analyzed for each genotype are indicated in brackets and significance levels are calculated by Wilcoxon rank-sum tests. The numerical data underlying this figure can be found in S1 Data. DKO, double knockout; M2KO, Mbnl2 knockout; M3KO, Mbnl3 knockout; qPCR, quantitative PCR. (TIF) [file pbio.3001615.s006.tif]

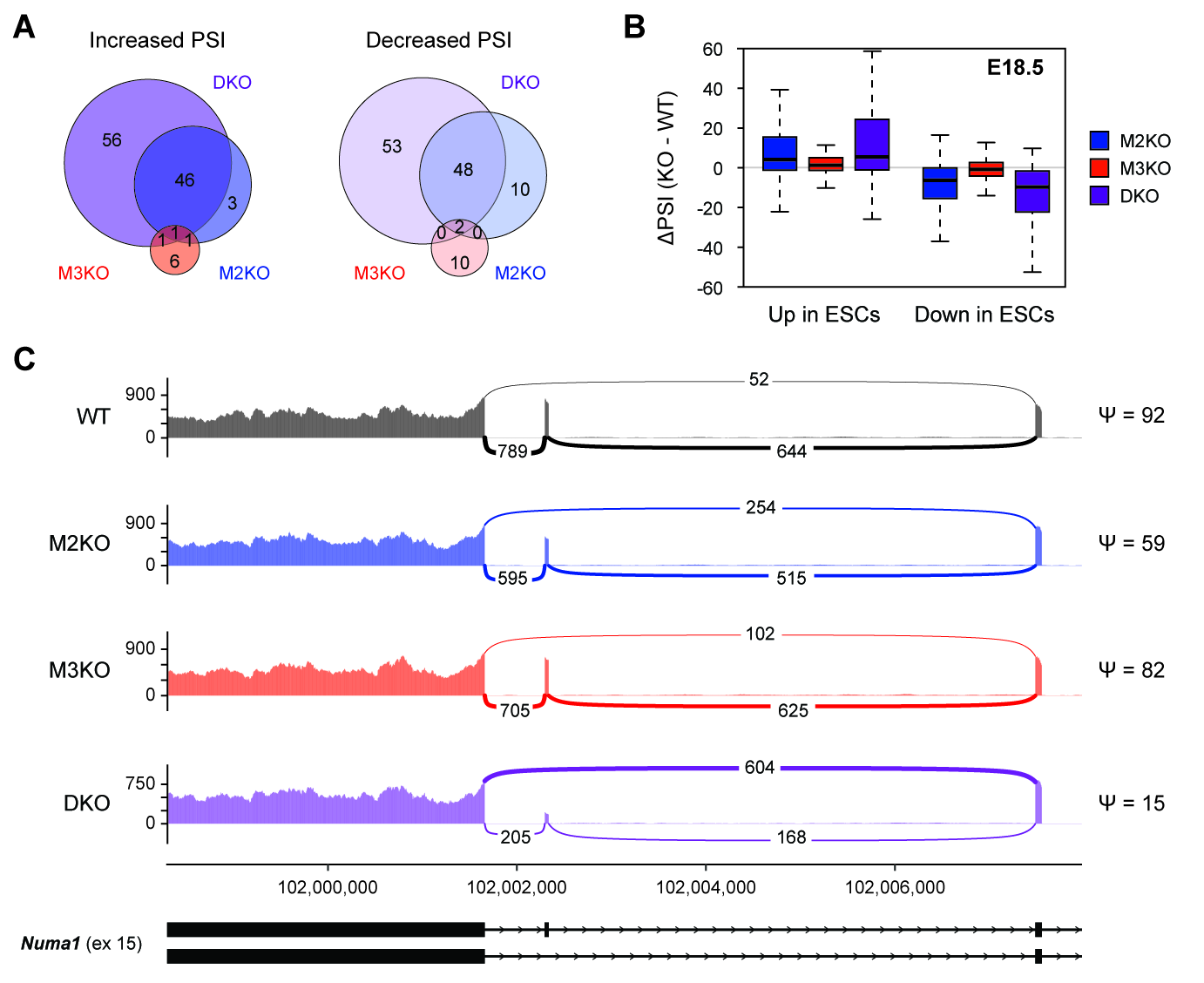

Supplement: S6 Fig — (A) Venn diagrams showing the overlap in differential spliced exons between M2KO, M3KO, and DKO placentas at E18.5. (B) Boxplots showing the change in PSI in the indicated KO versus WT placentas at E18.5 for all exons with differential inclusion between ESCs and differentiated cell and tissues. (C) Sashimi plots showing usage of Numa1 exon 15 in placentas with the indicated genotypes at E13.5. The PSI values for the exons in the different genotypes are shown to the right of the plots. The numerical data underlying this figure can be found in S1 Data. DKO, double knockout; M2KO, Mbnl2 knockout; M3KO, Mbnl3 knockout; PSI, percentage spliced in; WT, wild-type. (TIF) [file pbio.3001615.s007.tif]

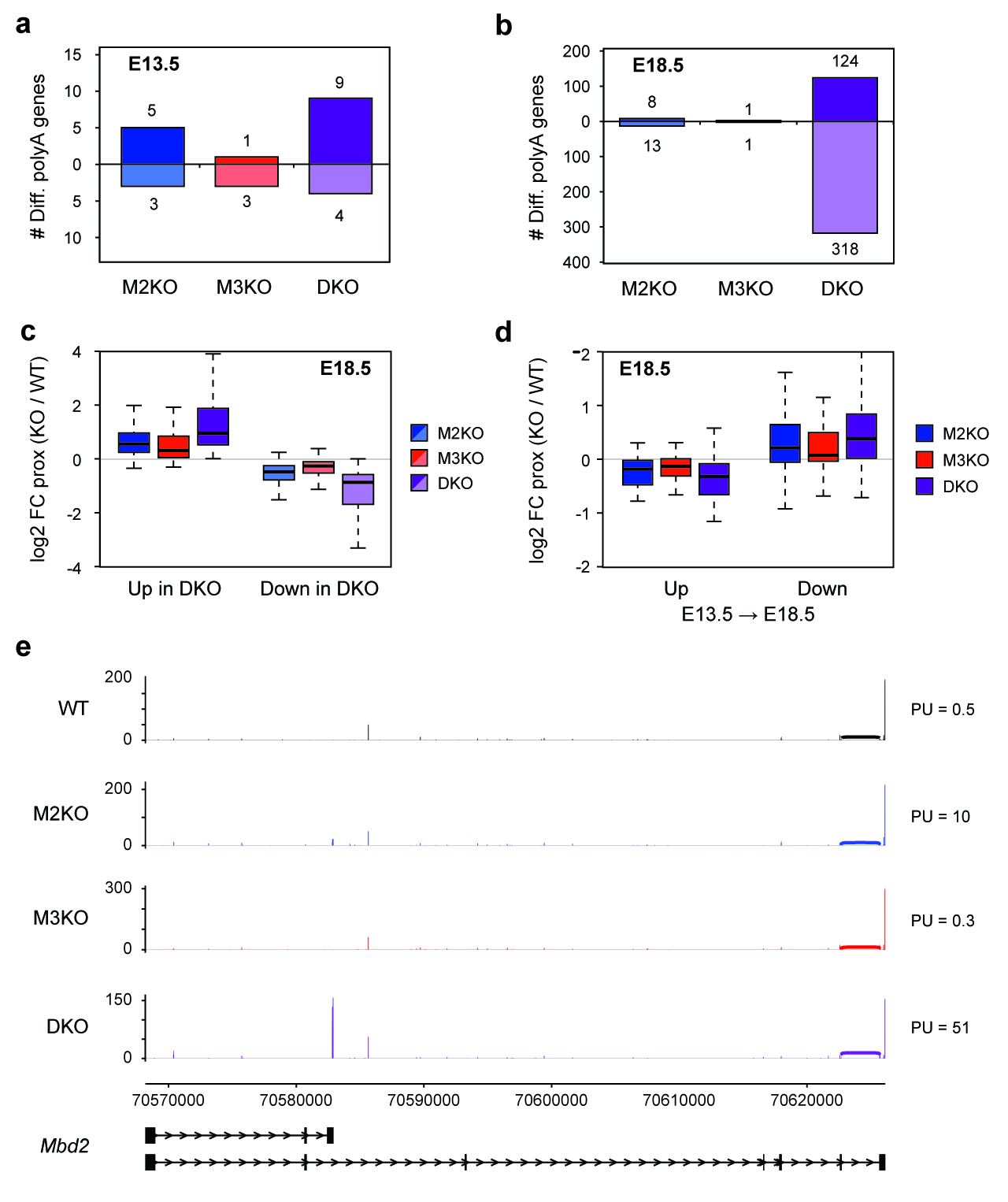

Supplement: S7 Fig — (A, B) Bar charts showing the number of genes with differentially used alternative polyadenylation (polyA) sites in M2KO, M3KO, and DKO placentas at E13.5 (A) and E18.5 (B). Genes whose proximal polyA site increased usage in KOs are tallied above the x-axis while genes with reduced proximal polyA usage are tallied below. (C) Boxplots showing the difference in proximal polyA site usage (log2 FC) in the indicated KOs versus WT placentas for all genes with differentially used polyA sites in DKOs versus WTs at E18.5. Up in DKO, N = 124; down in DKO, N = 318. (D) Boxplots showing the change in proximal polyA site usage in the indicated KO versus WT placentas at E18.5 for all genes polyA site pairs that are differentially used between E13.5 and E18.5 WT placenta. Up in E18.5, N = 31; down in E18.5, N = 77. (E) Sashimi plots showing usage of the 2 competing polyA sites in Mbd2 in placentas with the indicated genotypes at E13.5 based on 3′-seq data. The PU values for the proximal polyA site in the different genotypes are shown to the right of the plots. Coordinates correspond to mm10. The numerical data underlying this figure can be found in S1 Data. DKO, double knockout; KO, knockout; M2KO, Mbnl2 knockout; M3KO, Mbnl3 knockout; PU, percent of usage; WT, wild-type. (TIF) [file pbio.3001615.s008.tif]

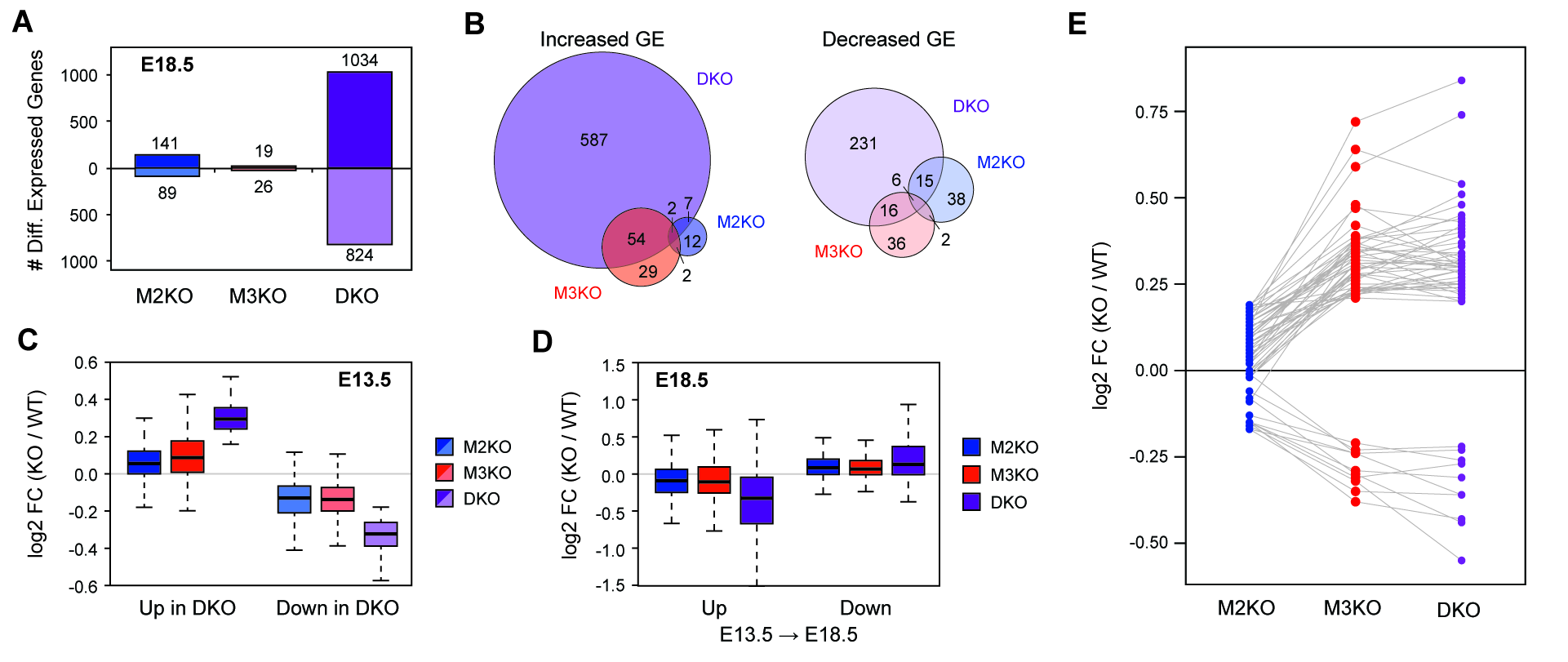

Supplement: S8 Fig — (A) Bar charts showing the number of differentially expressed genes in M2KO, M3KO, and DKO placentas at E18.5. Genes with increased expression in KOs are tallied above the x-axis, while genes with reduced expression are tallied below. (B) Venn diagrams showing the overlap in differentially expressed genes between M2KO, M3KO, and DKO placentas at E13.5. (C) Boxplots showing the difference in gene expression (log2 FC) in the indicated KOs versus WT placentas for all genes differentially expressed in DKOs versus WTs at E13.5. (D) Boxplots showing the change in expression in the indicated KO versus WT placentas at E18.5 for all genes differentially expressed between E13.5 and E18.5 WT placenta. (E) Dot plots showing the change in expression in the indicated KO versus WT placentas at E13.5 for genes that are differentially expressed only in M3KOs and DKOs. The numerical data underlying this figure can be found in S1 Data. DKO, double knockout; FC, fold change; M2KO, Mbnl2 knockout; M3KO, Mbnl3 knockout; WT, wild-type. (TIF) [file pbio.3001615.s009.tif]

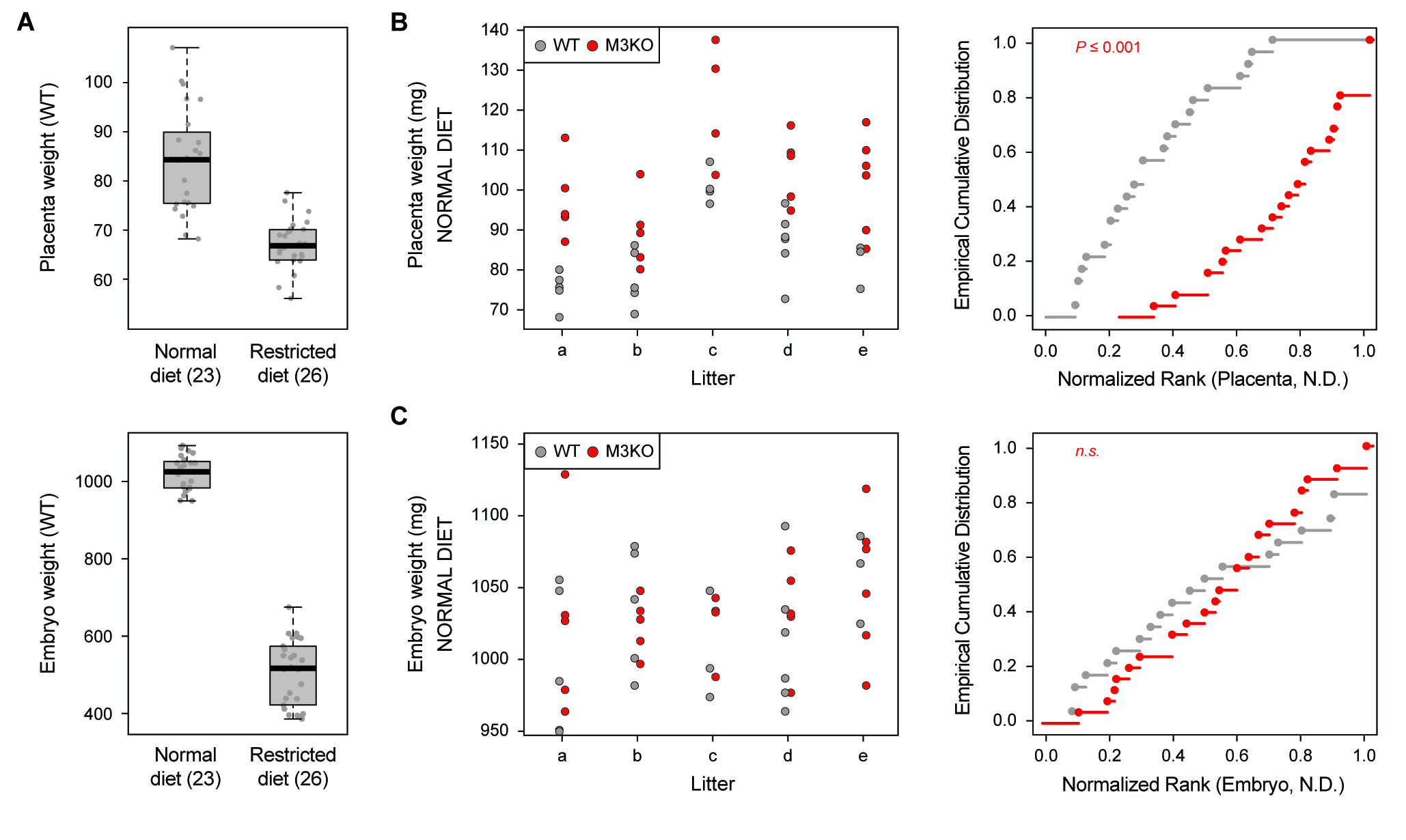

Supplement: S9 Fig — (A) Weight distributions of WT placentas (top) and embryos (bottom) under normal or calorie restricted diets. (B, C) Dot plots showing the weights of individual WT (gray) and M3KO (red, M3KO) placentas (B) and embryos (C) harvested from 5 mothers fed with N.D. and the corresponding empirical cumulative distribution plot for each genotype. Significance levels were calculated by a permutation test with 1,000 iterations swapping the genotype labels within each litter. The numerical data underlying this figure can be found in S1 Data. M3KO, Mbnl3 knockout; N.D., normal diet; WT, wild-type. (TIF) [file pbio.3001615.s010.tif]
