# Supplementary material for: The X-linked splicing regulator MBNL3 has been co-opted to restrict placental growth in eutherians
Source: PLoS Biol. 2022 Apr 27;20(4):e3001615. doi: 10.1371/journal.pbio.3001615 (PMC9084524; doi:10.1371/journal.pbio.3001615)
Supplement: S1 Raw images — (PDF) [file pbio.3001615.s027.pdf]

Western blot images used to generate Fig. S2d. Images of chemiluminescent western blots were captured directly using an Amersham Imager 600.

Mbnl3 blot

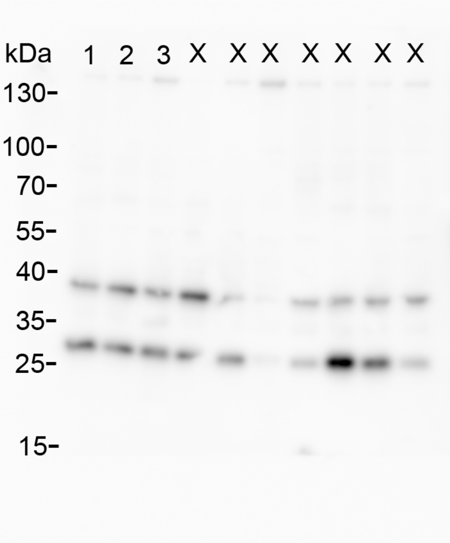

1) E11.5 placenta sample
2) E13.5 placenta sample
3) E18.5 placenta sample

Lanes not included in the final figure are marked with an X

Lamin B1 control blot (created by stripping and reprobing the Mbnl3 blot)

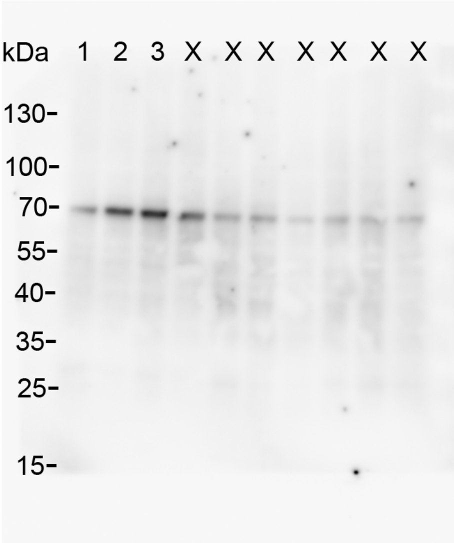

1) E11.5 placenta sample
2) E13.5 placenta sample
3) E18.5 placenta sample

Lanes not included in the final figure are marked with an X
