# Supplementary material for: The X-linked splicing regulator MBNL3 has been co-opted to restrict placental growth in eutherians
Source: PLoS Biol. 2022 Apr 27;20(4):e3001615. doi: 10.1371/journal.pbio.3001615 (PMC9084524; doi:10.1371/journal.pbio.3001615)
Supplement: Attachment — Submitted filename: Reviewer_answers-R1-v3.pdf [file pbio.3001615.s028.pdf]

REVIEWERS' COMMENTS:

Reviewer #1:
[identifies himself as Andrew Ward]

This is an excellent study of Mnbl3 gene function that uncovers its role in trophoblast and placental development, evolutionary changes in the regulation of the gene and in RNA-binding preference of MBNL3 protein, as well as potential molecular mechanisms of action. This adds up to a well-rounded and convincing story. The transcriptomic data reveals changes in Myc regulated gene expression that could make a major contribution to the Mbnl3 KO phenotype, though there are currently 'disconnects' in that there is no evidence Myc is directly regulated by MBNL3, and Myc regulated increases in cell proliferation are inferred but not proven. The paper contains a great deal of novel data and makes an important contribution in the fields of placental development, function and evolution. There are one or two pieces of data that appear to be missing, these and my other detailed comments and suggestions are presented below.

R: We thank the Reviewer for his support and the useful suggestions to improve our manuscript.

Introduction

Nice and concise, covering the relevant background.

Results

P4 and Fig 1, language a bit imprecise, e.g. tissue stages not given for placenta and non-placenta samples; it would be helpful to indicate that only splicing factors were plotted and how many (197 according to the Methods); Is it true to say "Expression enrichment in the trophoblast for the top candidates was confirmed by in situ hybridization analysis" or rather something along the lines that "Consistent with the enrichment analysis, expression each of the top candidates was readily detected in the trophoblast by in situ hybridization analysis"?

R: We have amended P4, which now indicates that 197 splicing regulators were examined as well as the source from which they were obtained.

The placenta data used for the analysis was collected at a number of different stages as was the data from non-placental tissues (which shows an amalgamation of data from hundreds of samples, as collected in vastdb.crg.eu); therefore, we believe it is not feasible to give tissue stages for placenta or non-placenta samples within the main text and/or figure legend. However, this information is available for all samples in Supplementary Table 1 (see “Description” column and additional details about the samples and associated studies can be obtained at the SRA webpage using the provided IDs).

We have rephrased “Expression enrichment in the trophoblast for the top candidates was confirmed by in situ hybridization analysis” it now reads “For the top candidates, trophoblast expression was confirmed by *in situ* hybridization analysis”.

P4-5, How has the trophoblast stem cell compartment been defined?

R: The trophoblast stem cell compartment was defined morphologically/anatomically as the region of the extraembryonic ectoderm adjacent to the epiblast at E6.5 and the chorionic hinge at E7.5. This has now been clarified in the text.

P5, "both full-length and truncated MBNL3 protein isoforms were present in mouse placenta by Western blot analysis (Fig. S2d)". It would be useful to know whether the MBNL3 antisera used to probe Westerns is known to recognise the C-terminus of the protein. This could be stated here or in the Methods or legend to Fig. S2d.

R: The anti-sera was raised against a 15-amino acid C-terminal peptide (Poulus et al. 2013). We have added this information to the figure legend.

P5 and Fig 2d, The transient mouse transgenic experiment is covered in a somewhat cursory fashion. Three putative promoter/enhancer fragments are mentioned in the Methods but the only data is a single LacZ stained embryo (representative of 4/10 examined, Fig 2d). I wonder if there is more to be learned from the full dataset? At the least the fragments could be better defined, perhaps alongside some bioinformatic analysis of conserved elements or hypersensitive sites.

R: We apologize for this confusion. For practical reasons, the three cloned fragments, which span the region highlighted in Figs. 2A and S2g, were ligated together to form a single final construct that was tested. We have clarified this in the Methods section. Regarding the remaining 6 embryos, they were negative for lacZ staining, so not particularly informative.

Following the Reviewer's suggestion, we have now added a new figure (S2g), showing a UCSC Genome Browser view displaying different ChIP-seq tracks obtained from E9.5 placenta and the associated ChromHMM-defined chromatin states. This shows an enrichment for active chromatin marks (H3K27ac and H3K4me3) over most of the tested region.

P5-7, I have no particular issues with the phylogenetic sequence analyses or the MBNL3 binding studies, where the conclusions seem entirely commensurate with the data. The group have extensive prior experience with the RNAcompete method but it should be noted as a caveat that the conclusions are dependent on this one method.

R: Whilst we agree with the fact that all our conclusions are based on a single *in vitro*

method is a caveat, it should be noted that a number of the binding motifs that we have obtained using RNAcompete are supported by other studies that use alternative methods for motif discovery. For instance, the GCA/U motif we find for mammalian Mbnl3 is very similar to the Mbnl3 motif identified in previous CLIP-seq based studies (Poulos et al. 2013; Sznajder et al. 2016; Taylor et al. 2018). Moreover, our findings that truncated MBNL isoforms lacking the first zinc finger pair (i.e. lacking ZFs 1 and 2) show reduced GCU binding preference is in agreement with previous work suggesting the binding preference of canonical MBNL proteins are largely driven by zinc finger 2 of the first zinc finger pair (Charizanis et al. 2012; Park et al. 2017; Hale et al. 2018). We have further clarified these points in the corresponding results section.

P7, "a modest [change] in…"?

R: This typographical error has been corrected; it should read "a modest increase".

P8, Both Mbnl3 and Mbnl2 are said to be expressed in trophoblastic tissues. A reference to Fig 1d would be useful here but also references to any information on their expression as the placenta develops. To what extent does their expression overlap in trophoblastic tissues through development?

R: We have added a reference to Fig. 1d and a further supplementary figure (S4) where we explore *Mbnl2* and *Mbnl3* expression in a recently published placental single-cell RNA sequencing dataset (Marsh and Blelloch 2020). In this analysis, we find *Mbnl2* and *Mbnl3* expression to be largely overlapping in the placenta, with both genes expressed in all placental cell subtypes at the stages examined (E9.5 to E14.5), although, at the later placental stages (from E10.5 onwards), *Mbnl2* does appear to be the predominant Mbnl factor in spongiotrophoblast cells.

P9, placental phenotypes are said to be due to Mbnl2/3 genes acting "partly redundantly in this tissue to ensure proper placental development". There is a lot of nice data presented on this but whether the genes act partly redundantly is not entirely clear (the trancriptomic data lends some support later on and perhaps this conclusion should be reserved for the Discussion). There seems to be an additive effect on placental size, with Mbnl2 KO causing size reduction and Mbnl3 an expansion. This adds up to a size similar to WT placentae in the double KO, but with the DKO placenta exhibiting a much more disorganised morphology than either single KO. This suggests the genes are acting largely independently of one another and perhaps while the placenta can compensate functionally for loss of either one, loss of both is more catastrophic.

R: We agree this was probably a premature juncture to state in the Results section that the genes act partly redundantly. Therefore, we have reserved this conclusion for the Discussion, as suggested. The arguments we put forward in support for the partial redundancy include: (i) the transcriptomic data (for example, see Fig. 4c, which shows that the same set of splicing events have small changes in inclusion in Mbnl3 KOs, moderate changes in M2KOs, and large changes in DKOs), (ii) the known regulatory

activities of the two proteins (both have been shown to be able to regulate the same splicing events but with different efficiencies; Taylor et al. 2018, Sznajder et al. 2016), (iii) the overlap in their placental expression patterns, and (iv) their protein sequence similarities. These data suggest that the genes can compensate functionally for one another and that it is the removal of this direct compensatory mechanism that results in the severe phenotype seen in the DKOs.

P9, in addition to the plots of embryo and placenta size (Fig. 3 and Fig S4) it may be useful to plot placental efficiency (fetal mass as a function of placental mass). This would highlight that although enlarged the Mnbl3 KO placenta is inefficient (see Discussion point below).

R: We have added these plots in Fig. S5f, and we now highlight the inefficiency of the Mbnl3 placenta in the text for this section.

P9, Inclusion of the conditional KO strategies is very nice but in both cases the placental weight data is missing. In the case of paternal transmission of Mnbl3 to produce Mnbl3+/p offspring, are the placentae large as in the homozygotes? In contrast, for the epiblast-specific KOs, was placental size roughly normal? Incidentally, here (including in Fig S4) the nomenclature could be tightened up as convention dictates that the alleles are written maternal/paternal and where it is relevant to indicate which parental allele is mutant this can be expressed as m/+ or +/p.

R: We have added placental weight data to Fig. S5h and j. Mnbl3$^{m/+}$ placentae show a clear size increase, similar that seen in the full *Mbnl3* KOs. Intriguingly, for *Mbnl2* epiblast-specific KOs, a decrease in placenta size is still seen, suggesting that this phenotype, at least in part, is not directly caused by loss of *Mbnl2* from the placenta. We have added a brief discourse about this to the Supplementary Discussion.

Additionally, we have amended the nomenclature for the conditional KOs, as requested, while keeping the nomenclature we have used for the full KOs throughout the manuscript (i.e. M2KO, M3KO and DKO when referring to constitutive KOs).

P9, "At both time points substantially more and larger splicing changes were seen in DKO than single KO placentas" and "number and extent of splicing changes"; I can see the evidence substantiates greater numbers of changes in DKO placentae but I am not seeing in addition a 'larger' or greater 'extent' of changes.

R: We are sorry for this confusion. Unfortunately, in the original manuscript the figure panels showing this phenomenon were not clearly indicated. Reference to the appropriate figure panels has now been added. Specifically, these are Fig. 4c, which shows the same set of splicing events have small changes in inclusion in *Mbnl3* KOs, moderate changes in *Mbnl2* KOs and large changes in DKOs, and that the changes occur in the same direction, and Fig. 4d-f and Fig. S6b-c where the extent of splicing changes can also be appreciated.

P10-11, there is a general problem in interpreting the transcriptomic data that is common to all KO experiments of this kind in that it is difficult to resolve changes that are a cause or consequence of the resulting phenotype. Logically, any causal changes should occur before the primary phenotype emerges. Here, transcriptomic data was collected at multiple stages with the earliest data from e11.5 placentae, at around the same time changes in Mbnl3 KO placenta size was first detected. This builds a picture that Myc-driven expansion of the labyrinth could underlie the primary phenotype in Mbnl3 KO placentas. A Myc:Mbnl3 double KO would help to pin this down conclusively, but may be difficult to perform because of the embryo lethal phenotype of Myc homozygous KOs. However, the case could be strengthened by addressing when and where MYC expression is increased in the trophoblast and through showing that there are associated increases in cell proliferation (increased proliferation is alluded to on P12 but not shown).

R: We agree the mechanistic link to Myc is largely circumstantial at this point. However, whilst we hope to resolve this in the future, given the substantial amount of work it would require to address these questions, and following the directions of the journal's editors, we feel it is outside of the scope of this study. We have now made it clearer in the Discussion that the mechanistic link with Myc is only putative at this stage.

Regarding the increases in proliferation, we have previously attempted to investigate this using Phospo-histone 3 staining but given the low mitotic index that we detected at E11.5 (~4%), the modest size increase seen in Mbnl3 KO placentas (~20%) and the level of variability both in terms of overall size and levels of mitosis seen in both WT and KO placentas it seemed unfeasible that we would detect significant changes in the mitotic index using this approach. For instance, Frank et. al. (2002) were unable to detect significantly increased proliferation (or reduced apoptosis) in Phlda2 KO placentas, which also show a relatively small size increase of around 25% using BrdUrd incorporation and staining with anti-Ki67 antibody, and DNA end-labelling assays. In the end, they attributed the net weight increase observed by mid-gestation in the Phlda2-null placentas to the cumulative effect of a small net difference in trophoblast proliferation, which they were unable to detect due to technical limitations.

P11, The following statement may need further thought: "Consistent with the X-linked nature of Mbnl3 and the expected resulting maternal bias as well as the increase in placenta size, amongst these genes were a number with a potential role in nutrient uptake, Slc2a3, LipG, Atp1b1, Steap3 and Slc19a2, which where all up-regulated following Mbnl3 loss". Once the placenta has become dysmorphic it seems likely that expression of genes involved in nutrient uptake regulation will compensate for these deficiencies, as has been shown in other KO models. Without evidence that MBNL3 directly regulates the indicated genes the evolutionary argument is weakened somewhat in this instance.

R: We have modified the text to acknowledge the possibility that these gene expression changes could also be due to compensation mechanisms. However, it should be kept in mind that if this were the case we would expect the level of up-regulation to be higher in

DKOs, which due to their reduced size vs Mbnl3 KOs and increased dysmorphia would be expected to trigger even stronger compensation.

P12, the lack of an increase in embryo growth in association with the expanded placental size, and labyrinthine volume in particular, is a bit surprising but not entirely without precedent (see for example global versus placenta-specific KO of Igf2). The inclusion of the maternal gestational caloric restriction experiment is therefore very interesting. The effect on embryo size is subtle and because of the way the data have been normalised it is only possible to see the impact of caloric restriction on embryo size in the box-plot of Fig S8a . It is not clear why there is no equivalent plot for placentae. More generally, this experiment throws up a number of questions that seem worthy of further study (e.g. what happens to nutrient uptake gene expression, do the embryo weight gains persist into post-natal life?). but I am not suggesting they necessarily should be a part of this paper.

R: We have added the box plot of placental size to Fig. S8 as suggested. We agree the results of this experiment raises a number of further questions; however, in agreement with the Reviewer, we believe answering them falls outside of the scope of this study.

Discussion

Arguments are generally concise and balanced. A lot of important discussion points are made in the Results section. Here there is some additional speculation of the molecular mechanism of MNBL3 action and on the evolution of Mnbl3 given its X-linked status. The letter point could perhaps be better made by alluding to placental efficiency, rather than size, and to think about the placenta contributing to producing a fetus that is of an optimal size at term. The authors might also draw on other examples of any other X-linked genes or maternally imprinted genes (e.g. Cdkn1c, Grb10, Igf2r) known to limit growth of the embryo and/or placenta.

R: We have significantly extended the part of the discussion related to the evolution of *Mnbl3* and the relationship of this to its X-linked status. In particular, we have added a section considering the effects of *Mbnl3* KO on placenta efficiency and putting this in the context of other described KOs of X-linked and autosomal paternally silenced genes.

Methods

P17, It is not clear what "(red box)" and "(green boxes)" refer to without a figure reference.

R: A figure reference has been added as suggested

Figure legends

S5, Include 'Numa1' in the title line.

R: The title line has been changed to include Numa1

Reviewer #2:

Spruce and colleagues present an exploration of the X-linked gene MBNL3 in the control of eutherian placental growth. The placenta is a transient organ that mediates maternal-offspring interactions and is therefore the site of conflict between maternally- and paternally-expressed genes, reflecting differences in the most effective reproductive strategies of males and females. As a consequence, the X chromosome - inherited matrilineally - is enriched for genes that play key roles in placental development and function.

Most studies examining the mechanisms of placental development and evolution have focused on the level of gene expression, but co- or post-transcriptional processing (splicing, polyadenylation) is likely to play an important role. The authors set out to test this hypothesis. They begin in an unbiased way by comparing the expression of known regulators of splicing between trophoblastic and embryonic tissues in mice, and identify Mbnl3 as being the most enriched regulator in the placenta. The authors then go on to examine the evolution of this gene and determine that it has acquired a novel function in the eutherian placenta. The study has implications for understanding: 1) how the placenta has co-opted ancestral genes to enable it to perform its unique functions in supporting embryonic development; 2) the molecular and genetic basis for that process; and 3) how the genome responds to conflict over resource allocation. The study has broad interest to the fields of embryonic development, evo devo, and the regulation of co-transcriptional processes.

The authors present an excellent manuscript. It is well-written and would carefully guide any reader who is not an expert in this area through the results and conclusions. The study itself is well-designed and the authors use an innovative combination of approaches to reach their conclusion. Throughout the paper, the authors develop hypotheses based on their observations and then test these empirically using a diverse set of tools including transgenic reporter assays and knockout mouse lines. They validate many of their key findings by independent methods - for example, RNA-seq experiments were validated by qRT-PCR on independent samples; observations from sequence data were validated by in situ hybridization and transgenic reporter assays; and results from systemic mouse KO models are validated by using a placenta-specific KO. Overall, the approaches used are thorough, rigorous and robust.

The weakest part of the paper is the association between Mbnl3 and Myc. The authors clearly realize this and do not over-interpret their findings. Although a mechanistic link between these two genes would strengthen the paper further, it is already a tour de force in evolutionary development, and I recognize that this is beyond the scope of the current manuscript.

I have only one very minor suggestion for the authors to consider. In Figure 2, it is not immediately clear which sets of data refer to the chimeric proteins. I suggest indicating in the figure which are the chimeric proteins, perhaps with a line underneath their labels to indicate this. As an alternative, providing a key to the naming of the proteins in the figure legend would help.

R: Thank you very much for your positive words about our study. We have now added an asterisk to the labels of the chimeras and refer to it in the figure legend.
